# COVID-19 impact on hospitalizations in older adults with chronic conditions: a real-world analysis from Lombardy, Italy

**DOI:** 10.1093/eurpub/ckac129.412

**Published:** 2022-10-25

**Authors:** C Bosetti, M Rognoni, R Ciampichini, M Scala, L Cavalieri d'Oro, A Zucchi, A Amerio, L Iacoviello, A Odone, S Gallus

**Affiliations:** 1 Department of Oncology, IRCCS Mario Negri Institute for Pharmacologic Research, Milan, Italy; 2 Brianza Health Protection Agency, Monza, Italy; 3 Bergamo Health Protection Agency, Bergamo, Italy; 4 DINOGMI, University of Genoa, Genoa, Italy; 5 IRCCS San Martino Polyclinic Hospital, Genoa, Italy; 6 Department of Epidemiology and Prevention, IRCCS Neuromed, Pozzilli, Italy; 7 EPIMED, Insubria University, Varese, Italy; 8 Department of Public Health, University of Pavia, Pavia, Italy; 9 School of Medicine, Vita-Salute San Raffaele University, Milan, Italy; 10 Department of Environmental Health Sciences, IRCCS Mario Negri Institute for Pharmacologic Research, Milan, Italy

## Abstract

**Background:**

Healthcare delivery reorganization during the COVID-19 emergency may have had a significant impact on access to care for older adults with chronic conditions.

**Methods:**

We investigated such impact among all adults with chronic conditions aged ≥65 years, identified through the electronic health databases of two local health agencies - ATS Brianza and ATS Bergamo - from the Lombardy region, Italy. We considered hospitalizations for 2020 compared to the average 2017-2019 and quantified differences using rate ratios (RRs).

**Results:**

Overall, in 2017-2019 there were a mean of 374,855 older adults with ≥1 chronic condition per year in the two ATS and 405,371 in 2020. Hospitalizations significantly decreased from 84,624 (225.8/1000) in 2017-2019 to 78,345 (193.3/1000) in 2020 (RR 0.86). Declines were reported in individuals with many chronic conditions and for most Major Diagnostic Categories, except for diseases of the respiratory system. The strongest reductions were observed in hospitalizations for individuals with active tumours, particularly for surgical ones. Hospitalization rates increased in individuals with diabetes, likely due to COVID-19-related diseases.

**Conclusions:**

Although determinants of the decrease in demand and supply for care among chronic older adults are to be further explored, this raises awareness on their impacts on chronic patients’ health in the medium and long run.

